# Associations between birth-related stressors, social reward responsiveness, and postpartum internalizing symptoms

**DOI:** 10.1080/10615806.2026.2620592

**Published:** 2026-03-30

**Authors:** Emilia F. Cárdenas, Maya Jackson, Julia Garon-Bissonnette, Kate L. Harkness, Kathryn L. Humphreys, Autumn Kujawa

**Affiliations:** a Department of Psychology and Human Development, Vanderbilt University, Nashville, TN, USA; b Department of Psychology, The Pennsylvania State University, University Park, PA, USA; c Department of Psychology, Queen’s University, Kingston, Canada

**Keywords:** Birth stress, social reward, postpartum depression, postpartum anxiety, postpartum internalizing symptoms

## Abstract

**Background and Objectives:**

Birth-related stressors and reward processing are associated with postpartum internalizing symptoms. We examined main effects and interactions of reward processing and birth-related stressors on postpartum internalizing symptoms.

**Design:**

Cross-sectional.

**Methods:**

Women (*n* = 95) completed the Birth Experience Interview and questionnaires at 8-weeks postpartum. In addition, while electroencephalogram (EEG) data were collected, participants completed a Social Incentive Delay task to measure neural responses to feedback indicating a correct response and receipt of a social reward (photo of own infant) vs. feedback indicating an incorrect response and neutral outcome. EEG analyses focused on reward positivity (RewP) to reward feedback, adjusting for responses to neutral feedback. Participants completed questionnaires on depressive, anxiety, and traumatic intrusion symptoms.

**Results:**

Greater birth-related severity and violations of expectations during childbirth were associated with more traumatic intrusion symptoms, but not depressive or anxiety symptoms. Surprisingly, an *enhanced* social RewP was associated with greater depression and anxiety symptoms. Social RewP moderated associations between birth experiences and postpartum traumatic intrusions; greater violation of expectations was associated with greater traumatic intrusions for those with relatively larger social RewP.

**Conclusions:**

Results support the utility of using dimensions of birth-related stressors as well as ERPs for measuring individual differences in reward processing relevant to postpartum internalizing symptoms.

## Introduction

Four million women give birth each year in the U.S. (Martin et al., 2018). Approximately 20% meet criteria for clinically significant postpartum internalizing symptoms (Gavin et al., 2005; Nakić Radoš et al., 2018; Yildiz et al., 2017). In mothers, postpartum internalizing symptoms are associated with functional impairments, severe distress, and disturbances in relationships with their infants (Pearlstein et al., 2013). These symptoms are also associated with a range of adverse outcomes for infants (Righetti-Veltema et al., 2003). Although birth-related stressors present as risk factors for postpartum internalizing symptoms, not all women who experience birth stress report postpartum internalizing symptoms. The identification of underlying processes driving vulnerability and resilience is needed to inform prevention efforts.

Well-established links exist between exposure to stressful events and depression and anxiety symptoms across the lifespan, including the postpartum period (Meyer et al., 2001). Birth-related complications are a common source of stress in the peripartum period, and nearly half of women giving birth endorse birth-related stressors (Bay & Sayiner, 2021). These stressors can include labor challenges, emergency interventions that violate expectations, and the need for intensive medical treatments (Goldenberg et al., 2008). Birth-related stressors can lead to lasting psychological and physical health problems for women (Simpson & Catling, 2016). Further, they may disrupt mothers’ affiliations with their infants and contribute to impaired child development (Simpson & Catling, 2016). Given the effects of birth-related stressors on the well-being of mothers and infants, research is required to identify moderators (i.e., vulnerability and resilience factors) that may influence health outcomes for new mothers.

No gold standard method exists to capture dimensions of birth-related stressors. Similar to measurements of general stress, many self-report instruments of birth-related stressors conflate exposure to the stressor with subjective experiences and symptoms (Ayers et al., 2018; Harkness & Monroe, 2016). These self-reported life stress measures are susceptible to several sources of bias (e.g., participants’ states and traits; Harkness & Monroe, 2016) and can thus be problematic when attempting to operationalize objective exposure to birth-related stressors. Contextual interview-derived instruments with independent ratings of stressful exposures allow researchers to evaluate exposures within the context of individuals’ circumstances and anchor these exposures to standardized ratings. This is the gold-standard in the assessment of stressful life events as it allows for greater precision and sensitivity in identifying exposure to stress independently of individuals’ stress perceptions (Harkness & Monroe, 2016). Thus, an important element of the current study is the use of a contextual interview-derived assessment to examine specific dimensions of birth-related stressors in association with internalizing symptoms, informing understanding of symptoms in the context of individuals’ circumstances (Harkness, 2013).

In addition to improved methods for assessing birth-related stressors, there is a need to better understand who is most susceptible to the effects of these stressors. The National Institute of Mental Health Research Domain Criteria (RDoC) initiative focuses on the study of dimensional constructs that integrate elements of psychology and biology to inform understanding of psychopathology. Internalizing disorders, including both anxiety and depression, have been characterized by alterations in RDoC’s reward responsiveness construct (Burkhouse et al., 2017). Reward responsiveness includes brain and behavioral responses to impending or possible reward (i.e., reward anticipation), the receipt of reward (i.e., initial response) and following repeated receipt of reward (i.e., reward satiation; National Institute of Mental Health, 2025). Outside of the peripartum period, neuroscience methods are commonly used to identify alterations in reward processing implicated in shaping responses to stress, including risk for depression, anxiety, and post-traumatic stress disorder (PTSD) symptoms (Kujawa & Burkhouse, 2017; Lieberman et al., 2017). There are some common patterns for reward responsiveness across these symptom domains, but there is also evidence of distinct alterations in each, raising the importance of considering symptom dimensions separately. Reward responsiveness is also thought to be impacted by stress exposure, including potentially serving as a mechanism by which stress influences risk of psychopathology and/or a moderator of effects of stress on psychopathology (Pegg & Kujawa, 2024).

Etiological models of internalizing symptoms outside of the peripartum period have focused on altered functioning in brain regions central to reward processing (Pizzagalli & Roberts, 2022). These models identify neural measures of reward system activation as potential biomarkers for internalizing symptoms (Ng et al., 2019). The associated processes, measured through electrical activity on the scalp (i.e., electroencephalography [EEG]), can be used to predict depression risk (Newson &Thiagarajan, 2019). Event-related potentials (ERP), a time-domain EEG scoring approach, provides information about the time course of emotional and cognitive processes at the neural level with millisecond precision (Sur & Sinha, 2009). ERPs can be used to identify biomarkers to further understanding of processes increasing risk for postpartum internalizing symptoms. One specific ERP used for the purpose of identifying risk for internalizing symptoms is the reward positivity (RewP) component. The RewP component emerges over frontocentral sites 250–350 ms after receiving reward compared to loss or neutral feedback and serves as an index of initial reward responsiveness.

Alterations in RewP have been consistently associated with internalizing symptoms in both youth and adults, and shown to moderate associations between stress exposure and symptoms. Prior studies have examined RewP in relation to internalizing symptoms, including both depression and anxiety, in adults (Burkhouse et al., 2016; 2017). In a sample of pregnant women, reduced RewP was found to be associated with greater antenatal depressive symptoms relative to other indicators of psychopathology risk (Mulligan et al., 2019). While blunted RewP has consistently been linked to depression across populations, findings with anxiety and PTSD are less consistent. For example, Kessel et al. (2015) found that children with higher generalized anxiety symptoms exhibited reduced responsivity to monetary gains versus losses, whereas children with higher social anxiety symptoms exhibited greater responsivity to monetary gains versus losses. Similarly, in a sample of adults, patients with higher distress and misery-based symptoms were characterized by a relatively blunted RewP, but RewP was unrelated to fear-based anxiety symptoms (Burkhouse et al., 2017). Although limited, there is also some evidence linking certain symptom dimensions of PTSD with the RewP. Specifically, Lieberman et al. (2017) found that hyperarousal symptoms, but not re-experiencing, were associated with enhanced RewP. These findings highlight the importance of considering multiple dimensions of internalizing symptoms in relation to RewP within the same sample. There is also evidence that individual differences in RewP moderate effects of stress on symptoms of psychopathology, particularly depression (Pegg & Kujawa, 2024).

Neural reward responsiveness is commonly measured in response to monetary reward feedback or anticipation (Novak & Foti, 2015). However, in the context of the postpartum period, social rewards may be particularly salient for new mothers. Research supports the role of reward-related brain function in the development and maintenance of caregiving behaviors (Swain et al., 2007). Specifically, in the postpartum period, new mothers have been found to display more activity in reward and motivation brain network (e.g., striatum, thalamus) when listening to the sounds of an infant cry relative to white noise (Lorberbaum et al., 2002), as well as when viewing images of their own infant versus an unknown child (Strathearn et al., 2008). There is also evidence for an association between mothers’ neural responses to infant faces and activation of the parental care system to provide protection (e.g., responding to infants in distress) and nurturance (e.g., viewing infants as affectively rewarding; Endendijk et al., 2020). Further, there is evidence that mothers without depression and anxiety, compared to mothers with elevated symptoms, exhibit increased reward-related neural responses to stimuli associated with their infants (Post & Leuner, 2019). Although versions of tasks traditionally using monetary rewards have utilized different types of social rewards (Nelson & Jarcho, 2021), we developed the first social reward task with social reward stimuli personally-salient to new mothers (Cárdenas, Jackson, et al., 2025). In addition, there is evidence suggesting social reward responsiveness is a vulnerability factor for depression in combination with stress exposure (Pegg et al., 2019); a combination of low RewP and stress is associated with greater symptoms of depression. However, the relationship between social reward responsiveness and birth-related stressors on postpartum internalizing symptoms in new mothers has yet to be investigated.

### This study

The goal of the current study was to refine the understanding of the roles of social reward responsiveness and birth-related stressors in postpartum internalizing symptoms in mothers. This study used a contextual interview to capture independently-rated birth-related stressors and a participant-specific reward task to capture reward responsiveness. First, we examined associations between social reward responsiveness and internalizing symptoms (i.e., postpartum depressive symptoms, anxiety, traumatic intrusions). We hypothesized reduced social RewP to reward feedback, indicating that the participant won a photograph of their own infants would be associated with greater symptoms, particularly of depression. Second, we tested birth-related stressors (i.e., overall severity, loss of control, violations of expectations) as predictors of internalizing symptoms. We hypothesized women with more marked birth-related stressors (i.e., overall severity, loss of control, violations of expectations) would endorse greater symptoms. Last, we tested social reward responsiveness as a moderator of associations between birth-related stressors and symptoms. We hypothesized a stronger association between birth-related stressors and symptoms for those who also exhibit low social reward responsiveness.

## Methods

### Participants

Pregnant people were recruited to participate in a study of predictors of peripartum depression and followed from approximately 20 weeks of gestation to 8 weeks postpartum. Eligibility criteria included: (a) pregnant and approximately 20 weeks of gestation at enrollment; (b) age between 18 and 40 years; (c) no previous diagnosis of mania/bipolar disorder, psychosis, or borderline personality disorders; (d) fluency in English. Participants were recruited through clinics in the community, online advertisements, flyers, and community events.

In total, 95 participants (*M_age_* = 31.21 years, *SD* = 4.31) completed the postpartum assessment and were included in the current analysis. All participants identified as women. In terms of race, 82% were White, 6% Black or African American, 9% Multiracial or Other race, 3% Asian. For ethnicity, 9% were Hispanic/Latinx. Most participants (85%) were married or in a domestic partnership, 12% were single and never married, and 3% divorced. For annual household income in US$, 1% reported $5001–$15,000, 6% $15,001–$30,000, 15% $30,001–$60,000, 19% $60,001–$90,000, 31% $90,001–$150,000, and 28% greater than $150,000. We used targeted advertisements and a broad recruitment strategy to oversample for postpartum depression risk. The Diagnostic Interview for Anxiety, Mood, and Obsessive–Compulsive and Related Neuropsychiatric Disorder (Tolin et al., 2018), a semistructured interview of DSM-5 disorders, was administered by research staff at each assessment visit to characterize clinical diagnoses. In this sample, 54% met criteria for a lifetime depressive disorder (i.e., major depressive disorder, persistent depressive disorder). The role of past depression is a well-established predictor of postpartum depression (Guintivano et al., 2018). At the time of enrollment, 51% of our sample met criteria for a past depressive disorder, 4% met criteria for a current depressive disorder, and 3% met criteria for both a past and current depressive disorder. During pregnancy, most participants (65%) reported no prior children, 26% had one child, 5% had two children, 1% had three children, and 3% had four children. Of those enrolled, 95 participants completed the 8-week postpartum assessment interview, and 91 also completed the EEG session.

### Procedures

Participants were recruited from a longitudinal study of women from the second trimester of pregnancy through 8 weeks postpartum. Written consent was obtained from all participants before study procedures began. The current analyses focus on measures collected at approximately 8 weeks postpartum. Participants completed a Zoom interview in which the Birth Experience Interview (BEI) was administered. After the BEI, they completed self-reported measures of depressive, anxiety, and traumatic intrusion symptoms. After the interview (*M* = 0.62 weeks, *SD* = 1.43), participants visited the lab to complete computer tasks, including the Social Incentive Delay task, during continuous EEG collection. The study was approved by the Vanderbilt University Institutional Review Board, and all procedures were in accordance with the ethical standards of the 1964 Helsinki Declaration and its later amendments.

### Task and measures

#### Birth-related stressors measure

Participants were interviewed using the BEI at the 8-week postpartum assessment to capture details of their birthing experience (Harkness et al., 2013). The BEI is based on the Life Events and Difficulties Schedule (Bifulco et al., 1989), an interview protocol that utilizes a contextual approach to independently assess the presence and severity of general life events. The semi-structured BEI was administered to elicit narratives about birth experiences (Harkness et al., 2013). After the interviews were completed, the interviewer constructed vignette summaries describing details of birth experiences that excluded any indicators of the participant’s emotional responses to events. The birth experience was defined as the period from when labor first began through 10–14 days postpartum. They then scored dimensions of birth-related stressors using a comprehensive manual of rating rules and standardized, anchored exemplars. Raters scored the BEI on three dimensions: overall severity, violation of expectations, and loss of control. Overall severity ratings reflected overall threat, or severity and were scored on a four-point Likert scale ranging from 5 (*little/none*) to 1 (*marked stressor*). Violation of expectations reflected the extent to which the birth deviated from participants’ birth plans. Raters scored violation of expectations on a four-point Likert scale ranging from 4 (*little/none*) to 1 (*marked stressor*). Loss of control reflected how much agency the mother had in decision-making. Raters scored loss of control on a four-point Likert scale ranging from 4 (*little/none*) to 1 (*marked stressor*). To facilitate interpretation of results, we transformed these values so that little/no stressor was the lower value (e.g., 1) and marked stressor was the higher value (e.g., 4). A second rater coded 69 of the 95 vignettes (73%) and derived their own ratings for the BEI dimensions. Intraclass correlation coefficient (ICC) estimates and their 95% confidence intervals were calculated based on a meanrating (*k* = 2), 2-way mixed-effects model. The ICC estimates for overall severity (ICC = .96, 95% CI = .93-.97), loss of control (ICC = .95, 95% CI = .93-.97), violation of expectations (ICC = .97, 95% CI = .95-.98) indicated excellent interrater reliability (see Koo & Li, 2016).

#### Social incentive delay task

**Figure 1.**
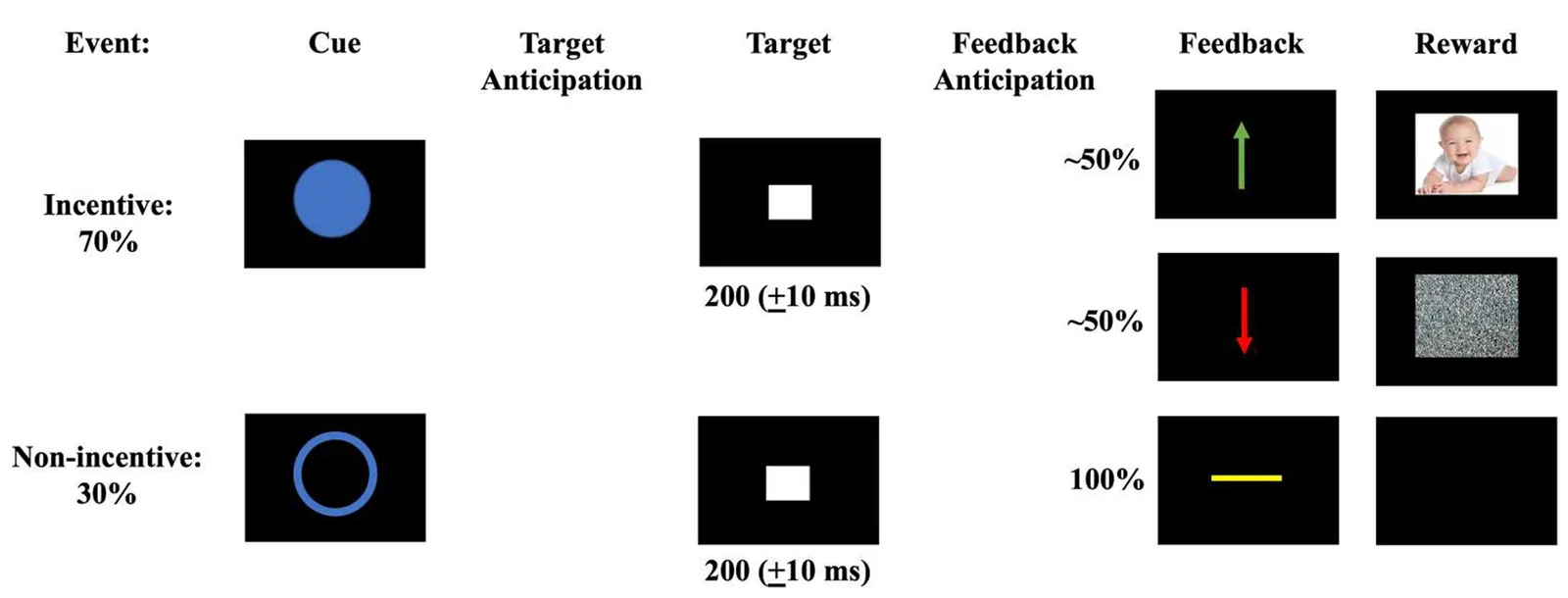
Social incentive delay task structure.

The Social Incentive Delay task (Figure 1) is a participant-specific social reward task developed for this study and adapted from the Monetary Incentive Delay task, a well-established task to investigate neural reward processing (Novak & Foti, 2015). At the start of each trial, participants observed a cue that indicated either a social incentive trial (i.e., blue circle) or a non-incentive trial (i.e., blue outline of a circle) for 500 ms. Following the cue, and as an anticipatory period, a fixation cross was presented for 2,000–2,500 ms. Then a white box was presented as a target. Participants were asked to respond to the target (i.e., pressing the left mouse button) as quickly as possible. The target presentation time was dynamically adjusted to achieve an approximately 50% success rate. The target presentation began at 200 ms and decreased by 100 ms following successful responses and increased by 10 ms following unsuccessful responses. Following the target presentation, a fixation cross was presented for a total of 1,500 ms from target presentation to feedback onset. On incentive trials, a green upward arrow appeared for responses to the target within the presentation period, indicating a correct response and that the participant would receive a reward. The green upward arrow was followed by a fixation cross for 1,000 ms, followed by a photograph of their infant (social reward) for 2,000 ms. A red downward arrow appeared for responses outside of the presentation period, indicating the response was too slow and they would receive a neutral image instead. The red downward arrow was followed by a fixation cross for 1,000 ms, followed by a photograph of rocks for 2,000 ms, which had previously been used as neutral stimuli for comparison to more appetitive images (Mühlberger et al., 2017). On non-incentive trials, a yellow dash was presented after the target on all trials, indicating that participants would not view a photograph regardless of reaction time. The yellow dash was followed by a fixation cross for 1,000 ms and then by a blank screen for 2,000 ms. A fixation cross for 1,000 ms was presented before the next trial.

#### Internalizing symptoms measure

Participants completed the Inventory of Depression and Anxiety Symptoms (IDAS), a validated measure of recent depressive and anxiety symptoms (Watson et al., 2007). The IDAS produces several empirically derived subscales for specific facets of internalizing symptoms. Given our focus on depression and responses to stressors and traumas related to birth, we focuses analyses on general depression and traumatic intrusions. The IDAS general depression scale has 20 items. Participants indicated the extent to which they experienced symptoms in the past two weeks on a scale from 1 (*not at all*) to 5 (*extremely*). The IDAS Traumatic Intrusions subscale, a 4-item subscale, was used to assess intrusive thoughts about trauma. Because the IDAS did not include a more general anxiety subscale, participants completed the Generalized Anxiety Disorder Assessment (GAD-7; Spitzer et al., 2006). This is a 7-item self-report questionnaire used as a screening tool and a measure for the level of generalized anxiety.

Four participants endorsed general depression scale scores above the screening cutoff for possible major depressive disorder. Three participants endorsed traumatic intrusion scores above the screening cutoff for possible PTSD. Ten participants endorsed generalized anxiety disorder above the cutoff used to identify potential clinical anxiety. Internal consistency (Cronbach’s alpha) was .87 for the IDAS general depression scale, .65 for the IDAS Traumatic Intrusions subscale, and .89 for the GAD-7. Reliability of the traumatic intrusions subscale fell within the questionable reliability range but was consistent with the range of values reported by Watson et al. (2007) in the development of the IDAS.

#### EEG data acquisition and processing

**Figure 2.**
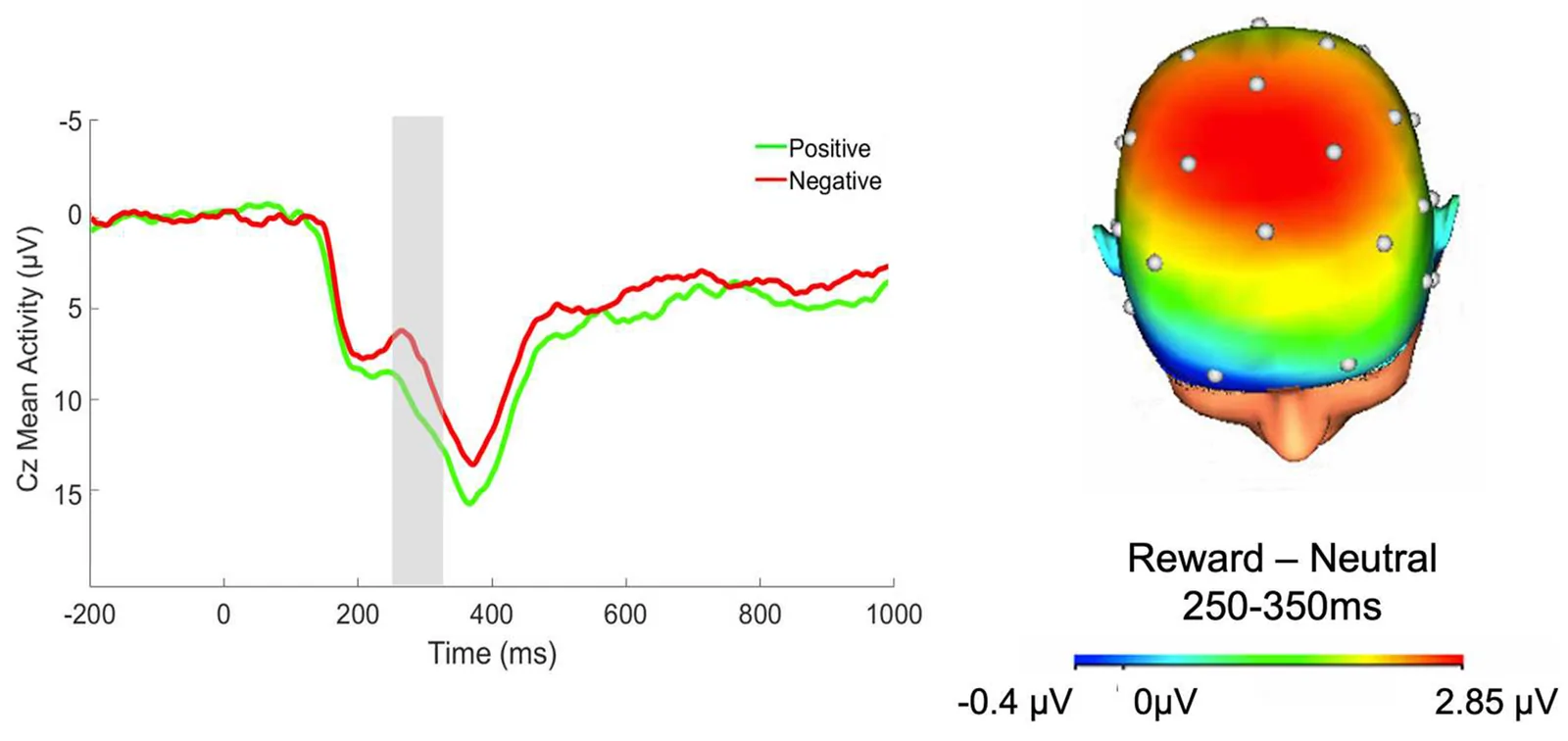
Grand averaged social RewP waveform, social RewP waveform and scalp topographies for responses to reward feedback minus responses to neutral feedback 250–350 ms. Note: The RewP was scored at Cz.

EEG data were recorded with a 32-electrode BrainProducts actiCHamp system (Munich, Germany) based on the standard 10/20 layout. Of note, a 32-channel cap was used for all participants, but at the beginning of the study, we focused only on 16 channels in applying gel to minimize time in close contact early in the COVID-19 pandemic (Simmons & Luck, 2020).^1^ BrainVision Analyzer software (Munich, Germany) was used to process EEG data using best practices (Luck et al., 2000). Impedances were reduced to 30 kΩ. A sampling rate of 1,000 Hz was used to digitize recordings. Data were re-referenced to linked mastoid electrodes and band-pass filtered with 0.1 and 30 Hz as cutoffs. Data was segmented −500 ms before and 1,000 ms after stimulus presentation. Ocular correction was conducted with modification of Gratton’s algorithm (Gratton et al., 1983). Vertical (VEO) and horizontal (HEO) electrooculogram were monitored bipolarly from sites above and below, as well as beside the outer canthus, of participants’ eyes. Due to modified COVID protocols, we did not apply facial electrodes for participants at the beginning of the study. For these participants, we used FP1 in lieu of VEO and FT9 and FT10 in lieu of HEO.^2^ Our prior research indicated that the mean amplitude of RewP does not significantly differ when using scalp vs. facial electrodes for ocular correction (Pegg et al., 2024). Automatic artifact rejection criteria was a voltage step greater than 50 μV between sample points. The maximum voltage difference between points was capped at 175 μV for trials, and the minimum voltage difference parameter was 0.5 μV within 100 ms intervals. After automatic artifact rejection, the data were inspected visually to reject any remaining artifacts. RewP was averaged across each condition, and baseline corrected 200 ms to 0 ms before feedback onset. Analyses focused on RewP, which is often scored between 250–350 ms at Cz (Mulligan et al., 2019; Pegg et al., 2019). This decision was also based on visualization of sample grand averages (Figure 2). All analyses focused on the unstandardized residual for the RewP for social reward feedback (i.e., green arrow), adjusting for the RewP to neutral feedback (i.e., red arrow; Meyer et al., 2017).

For the social incentive delay task, 8 of the 91 participants who completed the task were excluded for poor data quality and 1 was excluded because of technical issues. There was available EEG data for the remaining 82 participants. Following artifact rejection, included participants had on average 23.52 segments (*SD* = 2.54, range = 17–28) for the reward feedback condition and on average 24.65 segments (*SD* = 3.02, range = 15–33) for the neutral feedback condition. RewP split-half reliability was excellent for both RewP to reward feedback (Spearman-Brown coefficient: *rSB* = .89) and neutral feedback (Spearman-Brown coefficient: *rSB* = .87). We calculated unstandardized residual to RewP for reward feedback, adjusting for RewP to neutral feedback (Meyer et al., 2017) and used the RewP residual in all analyses.

### Data analytic plan

Grubb’s test with two tails is calculated for all variables to probe for outliers. Bivariate correlations quantified relations among variables. Results of Little’s MCAR test were null (*p* = .381). A non-significant *p*-value means that we cannot reject the hypothesis that the data are missing completely at random. Thus, we used full information maximum likelihood (FIML) in regression analyses via the lavaan package (Rosseel, 2012) in R (R Core Team, 2022). To examine associations between social reward responsiveness and postpartum symptoms, as well as associations between birth-related stressors and postpartum symptoms, we used bivariate correlations. To test social reward responsiveness as a moderator of associations between birth-related stressors and postpartum symptoms, we used multiple regression analyses with an interaction term to model the association between birth-related stressors and internalizing symptoms at different levels of social reward responsiveness. We also probed the regions of significance using an open source web calculator for simple slopes and regions of significance for MLR 2-way interactions (Preacher et al., 2006).

## Results

Table 1 presents the descriptive statistics for all study variables. Table 2 presents bivariate correlations, and associations are depicted below. Using Grubb’s test, an outlier was identified for loss of control and traumatic intrusions. The associated cases were retained for analysis.

### Bivariate correlations

Surprisingly, a larger social RewP was associated with more depressive and anxiety symptoms. However, social RewP was not statistically significantly associated with traumatic intrusion symptoms (Table 2).^3^ Birth-related stress dimensions were not statistically significantly associated with anxiety or depressive symptoms; however, greater overall severity and violations of expectations were statistically significantly associated with higher levels of traumatic intrusions (Table 2).

### Regression analyses

**Figure 3.**
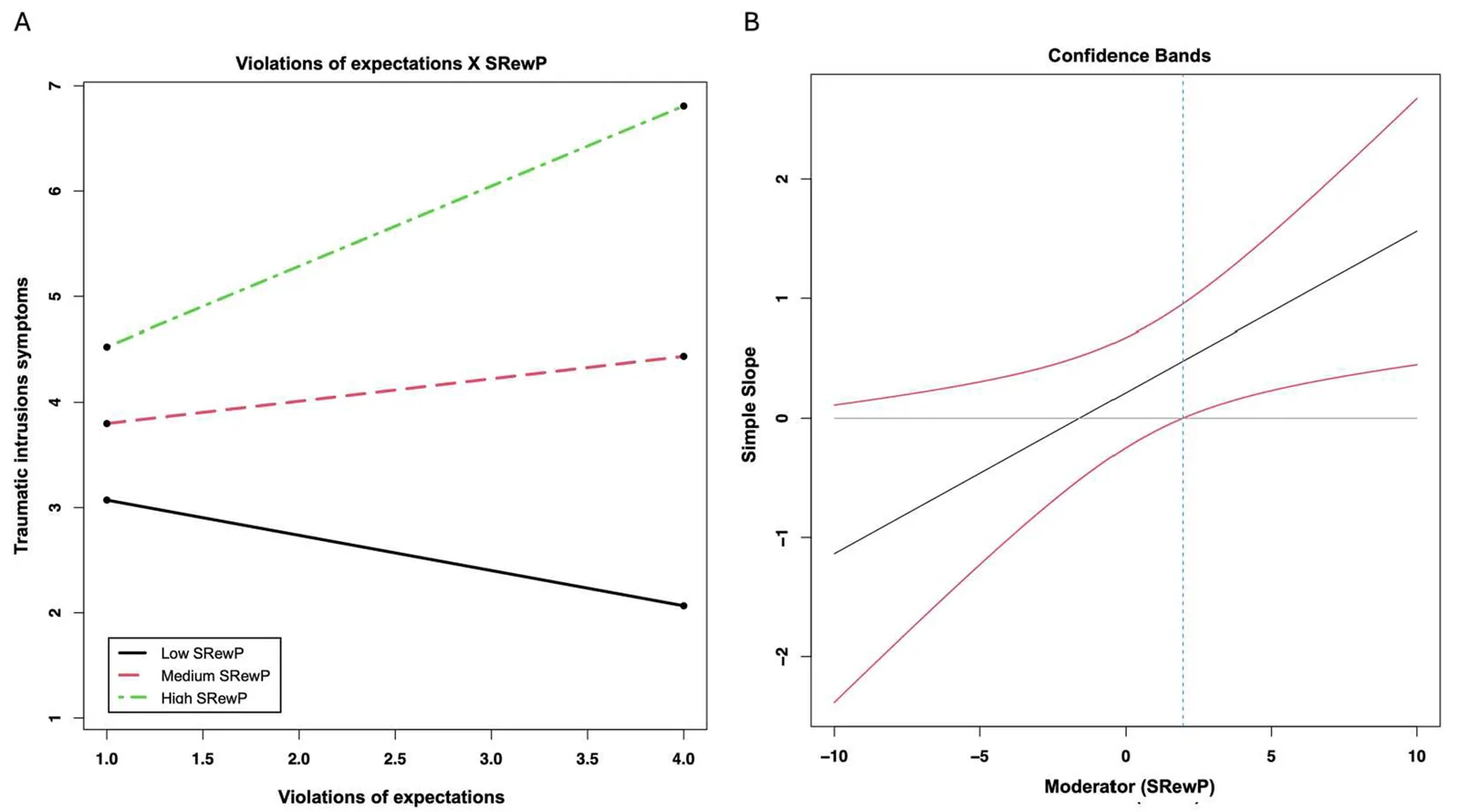
(A) Association between violations of expectations and traumatic intrusions at low, medium, and high levels of social RewP. (B) Associations between violations of expectations and traumatic intrusions as a function of social RewP.

Finally, three multiple regression models were tested to examine the interaction effects of social RewP and dimensions of birth-related stressors on the three symptom outcomes. The interaction between birth-related stressors and social RewP was not statistically associated with postpartum depressive symptoms (Table 3). There was also no statistically significant interaction when anxiety symptoms were examined as the outcome (Table 4). However, a statistically significant violation of expectations X social RewP interaction was found for traumatic intrusion symptoms (Table 5).^4^ Violations of expectations were associated with greater traumatic intrusions for those who had an enhanced social RewP (i.e., 84th percentile, *b* = 0.76, *SE* = 0.30 *p* = .012), but not average (i.e., 50th percentile, *b* = 0.21, *SE* = 0.23 *p* = .362) or blunted social RewP (i.e., 16th percentile, *b* = −0.34, *SE* = 0.35 *p* = .335; Figure 3(a)). The region of significance indicated a significant association between violations of expectations during birth (the focal predictor) and traumatic intrusion symptoms (the outcome) when social RewP (the moderator) was greater than 1.96 μV (Figure 3(b)).

## Discussion

In this study, we examined associations between social reward responsiveness, birth-related stressors, and internalizing symptoms in the early postpartum period for new mothers. A few key findings emerged, several of which were unexpected based on our hypotheses. First, an enhanced social RewP was associated with depressive and anxiety symptoms, but not traumatic intrusions. Second, there were no significant associations between birth-related stressors and depressive or anxiety symptoms, but greater overall severity and violations of expectations were associated with greater traumatic intrusion symptoms. Finally, social RewP moderated associations between violation of expectations and traumatic intrusion symptoms, such that violations of expectations were associated with greater traumatic intrusions for those who had a larger social RewP.

We tested social reward responsiveness as a moderator of the association between birth-related stressors and internalizing symptoms, given prior evidence that social RewP moderates effects of lifetime social stress exposure on depressive symptoms outside of the peripartum period (Pegg et al., 2019). There were no interactions between birth-related stressors and social RewP on postpartum depressive or anxiety symptoms. However, social RewP moderated associations between violations of expectations and traumatic intrusion symptoms at high levels of RewP. Specifically, participants with enhanced responses to rewards showed a greater risk for endorsing postpartum traumatic intrusions in response to the violation of expectations.

Our depression results contrast prior stress and depression research in which reduced neural responsiveness to reward has been associated with greater symptoms following stress exposure (e.g., Pegg et al., 2019). This could be because people with an elevated reward responsiveness, particularly for social rewards salient to the developmental period, may tend to expect more positive outcomes and potentially have more difficulty adjusting when expectations are thwarted. Although limited, there is some prior evidence linking certain symptom dimensions of PTSD (i.e., re-experiencing symptoms) with enhanced RewP (Lieberman et al., 2017). In addition, specific types of traumatic experiences have been linked with differential reward activation. Hendrikse et al. (2022) found that childhood abuse, but not neglect, was associated with increased reward-related activation in the ventral striatum in healthy adults. Furthermore, Letkiewicz et al. (2024) found that severe sexual abuse in childhood was associated with a heightened response to reward (i.e., larger RewP) in adulthood. Future research could include measures of additional PTSD symptom clusters to better understand associations with reward responsiveness, as well as a more thorough assessment of lifetime stress exposure that could impact reward circuitry.

Contrary to our hypothesis, we observed that greater social RewP was associated with both greater depressive and anxiety symptoms. This was surprising because most of the literature supports associations between lower RewP and greater depressive symptoms (Novak & Foti, 2015; Pegg et al., 2019). Our unique findings with social RewP may be related to the personally salient social rewards we used in the current study. Some EEG studies find that social rewards compared to monetary rewards may evoke a stronger neural response for individuals with depressive and anxiety symptoms (Nelson & Jarcho, 2021). Specifically, neural responses to social dislike feedback were associated with social anxiety. Findings suggest that different modalities of reward, such as receiving social dislike feedback from gender-matched peers, may provide specific insight and yield differential associations. Personalized social rewards can increase participant motivation and engagement. Alternatively, our findings on associations between social RewP and internalizing symptoms could be driven by either traumatic intrusions or anxiety symptoms, as these commonly co-occur with depression. There is prior evidence of increased RewP with some forms of anxiety (Kessler et al., 2015). It is important to note also that some symptoms of anxiety and depression can overlap with typical postpartum experiences (e.g., changes in sleep patterns and appetite; Saxbe et al., 2018). Thus, our focus on social reward and the cross-sectional nature of this study may suggest that a high level of motivational salience of infant cues may underlie internalizing symptoms during the early postpartum period. Additional research should further explore whether anxiety symptoms, or specifically certain typical postpartum experiences, better account for these findings.

Finally, this study is unique in that we used an interview-derived approach to assess birth-related stressors. Surprisingly, null associations were observed between birth-related stressors with postpartum depressive and anxiety symptoms. However, we did find correlations between both greater overall severity and violations of expectations with traumatic intrusion symptoms. There is prior evidence linking birth-related stressors with increased postpartum internalizing symptoms (Cárdenas, Yu, et al., 2025). Unexpectedly, the associations between birth-related stressors and postpartum depressive and anxiety symptoms were not significant in this study. However, we did observe an association with some dimensions of birth-related stressors (i.e., overall severity, violations of expectation) with traumatic intrusion symptoms. Birth-related experiences may be more strongly linked to facets of PTSD symptoms like traumatic intrusions than depressive symptoms more broadly. Extensions to samples with greater variability in birth experiences and risk factors is needed. Future research could include measures of additional PTSD symptom clusters to better understand associations with reward responsiveness, as well as a more thorough assessment of lifetime stress exposure that could impact reward circuitry.

### Limitations and future directions

There are several limitations. First, because of the cross-sectional nature of this study, we cannot determine the temporal associations between alterations in RewP underlying vulnerability to symptoms or as a manifestation of current symptoms. Second, although we were intentional about ethnic and racial diversity in our recruitment efforts, we had a lack of representation of minoritized racial and ethnic groups. Although results were consistent accounting for age/ethnicity, we were limited in power to test moderation by specific identities and cultural backgrounds. In addition, we were unable to comprehensively examine other factors that could impact health, like socioeconomic status. Underrepresentation of Black women is particularly problematic given established health disparities related to birth experiences (Singh, 2020), and extension of this work to larger and more diverse samples is critically needed. In addition, an outlier was identified for loss of control and traumatic intrusions. Results indicating relatively low levels of endorsement for birth experiences and symptoms suggest the need for replication in more high-risk samples. The traumatic intrusions scale was also relatively low in internal consistency. Lastly, although results support the possible utility of psychophysiology in elucidating risk factors for peripartum psychopathology, more research is necessary before such measures can be applied clinically. Additional research needs to examine the incremental utility of these measures above established protocols for screening women for peripartum psychopathology. Specifically, this research should be extended to clinical samples, as results may differ from those found in community samples.

## Conclusion

In the current study, enhanced social neural reward responsiveness was identified as potentially relevant in the emergence of postpartum internalizing symptoms. Contrary to our expectations noted in the introduction, we observed in bivariate associations that greater social reward responsiveness was associated with greater depressive and anxiety symptoms, but null for traumatic intrusion symptoms. Further, findings suggest that elevated social reward responsiveness may be associated with the presence of traumatic intrusion symptoms following violations of expectations in birth experiences. The findings highlight the potential utility of ERP measures of social reward responsiveness for clarifying pathways in postpartum psychopathology. Finally, the dimensional measure of birth-related stressors allowed for understanding of specific birth-related experiences most strongly related to psychopathology risk.

## Figures and Tables

**Table 1.** Descriptive statistics of study variables.

Variable	*n*	Mean	SD	Min–Max	Range	Skewness	Kurtosis
Depressive symptoms	94	36.59	8.64	23.00–65.00	42.00	0.95	0.83
Anxiety symptoms	94	3.68	3.97	0.00–16.00	16.00	1.40	1.19
Traumatic intrusion symptoms	94	4.98	1.57	4.00–11.00	7.00	1.73	2.61
BEI: Overall severity	95	2.48	1.00	1.00–5.00	4.00	0.21	−0.48
BEI: Loss of control	95	1.33	0.69	1.00–4.00	3.00	2.03	3.10
BEI: Violation of expectation	95	2.11	0.94	1.00–4.00	3.00	0.10	−1.30
Social RewP UR	82	0.00	4.06	−10.45–10.57	21.03	0.03	0.32

Note: BEI = Birth Experience Interview. UR = unstandardized residual.

**Table 2.** Bivariate correlations among study and exploratory variables.

Variable	1.	2.	3.	4.	5.	6.	7.
1. Depressive symptoms	1.00	.					
2. Anxiety symptoms	**.76	1.00					
3. Traumatic intrusion symptoms	**.41	**.52	1.00				
4. BEI: Overall severity	.07	.05	*.25	1.00			
5. BEI: Loss of control	<.01	.17	.16	**.67	1.00		
6. BEI: Violation of expectations	.10	.13	*.28	**.18	**.35	1.00	
7. Social RewP UR	*.25	*.26	.14	−.01	−.10	−.02	1.00

Notes:

* Correlation was significant at the .05 level (2-tailed).

** Correlation was significant at the .01 level (2-tailed). BEI = Birth Experience Interview. UR = unstandardized residual.

**Table 3.** Main and interactive effects of social RewP and birth-related stressors on postpartum depressive symptoms (*n* = 95).

Variable	*B*	SE	*b*	*p*	95% CI	
					*LL*	*UL*
Social RewP	1.57	0.92	0.73	.089	−0.24	3.38
BEI: Overall severity	0.79	1.18	0.08	.504	−1.53	3.10
BEI: Loss of control	1.72	1.36	0.14	.207	−0.95	4.39
BEI: Violation of expectations	−0.87	1.34	−0.08	.518	−3.50	1.76
BEI: Overall severity X Social RewP	−0.38	0.43	−0.47	.381	−1.21	0.46
BEI: Loss of control X Social RewP	−0.35	0.42	−0.22	.401	−1.16	0.46
BEI: Violation of expectations X Social RewP	0.15	0.34	0.15	.662	−0.51	0.81

Notes: *B* = unstandardized beta coefficient. b = standardized beta coefficient. LL = lower limit. UL = upper limit. BEI = Birth Experience Interview.

**Table 4.** Main and interactive effects of social RewP and birth-related stressors on postpartum anxiety symptoms (*n* = 95).

Variable	*B*	SE	*b*	*p*	95% CI	
					*LL*	*UL*
Social RewP UR	0.96	0.41	0.97	.019	0.16	1.76
BEI: Overall severity	−0.11	0.52	−0.03	.835	−1.14	0.92
BEI: Loss of control	0.89	0.61	0.16	.145	−0.31	2.09
BEI: Violation of expectations	0.40	0.59	0.10	.501	−0.76	1.56
BEI: Overall severity X Social RewP	−0.26	0.19	−0.69	.177	−0.63	0.12
BEI: Loss of control X Social RewP	−0.19	0.18	−0.26	.316	−0.55	0.18
BEI: Violation of expectations X Social RewP	0.10	0.15	0.22	.507	−0.19	0.39

Notes: *B* = unstandardized beta coefficient. *b* = standardized beta coefficient. LL = lower limit. UL = upper limit. Statistically significant findings were bolded. UR = unstandardized residual. BEI = Birth Experience Interview.

**Table 5.** Main and interactive effects of social RewP and birth-related stressors on postpartum traumatic intrusion symptoms (*n* = 95).

Variable	*B*	SE	*b*	*p*	95% CI	
					*LL*	*UL*
Social RewP UR	0.04	0.16	0.10	.781	−0.27	0.36
BEI: Overall severity	0.26	0.20	0.16	.211	−0.14	0.65
BEI: Loss of control	0.26	0.24	0.12	.275	−0.21	0.73
BEI: Violation of expectations	0.21	0.23	0.13	.359	−0.24	0.67
BEI: Overall severity X Social RewP	−0.08	0.07	−0.56	.269	−0.23	0.06
BEI: Loss of control X Social RewP	−0.04	0.07	−0.14	.567	−0.18	0.10
BEI: Violation of expectations X Social RewP	0.14	0.06	0.79	.018	0.02	0.25

Note: *B* = unstandardized beta coefficient. *b* = standardized beta coefficient. LL = lower limit. UL = upper limit. Statistically significant findings were bolded. UR = unstandardized residual. BEI = Birth Experience Interview.

## Data Availability

A subset of the data can be found on the NIMH Data Archive https://doi.org/10.15154/p6qb-av93. Other data are available by request to the corresponding author.

## Footnotes

1. Independent samples *t*-tests were conducted to test differences in RewP unstandardized residuals for participants with 32 (*n* = 75) vs. 16 (*n* = 7) electrodes. There was no statistically significant difference: Cohen’s *d* = 0.38, *p* = .341.

2. Independent samples *t*-tests were conducted to test differences in RewP unstandardized residuals for participants in which we used HEO and VEO (*n* = 53) vs. FP1 and FT9/FT10 (*n* = 29) for ocular correction. There was no statistically significant difference: *t*(80) = −0.78, *p* = .440; Cohen’s *d* = 0.18.

3. We examined depressive symptoms, traumatic intrusion symptoms, and generalized anxiety symptoms as unique predictors of social RewP in a multiple regression model. Although none of the associations were significant, the effect of generalized anxiety symptoms (*b* = 0.23; *SE* = 0.18; *p* = .195) appeared somewhat stronger than both depressive symptoms (*b* = 0.021; *SE* = 0.08; *p* = .791), and traumatic intrusions (*b* = 0.01; *SE* = 0.33; *p* = .968).

4. We examined age and racial/ethnic minority identify (dichotomized as white and non-Hispanic or another racial identity and/or Hispanic) as covariates in our three regression models. Key findings remained consistent, with a significant interaction between violations of expectations and social RewP on traumatic intrusions.
